# Body Mass Index and the Risk of Rheumatoid Arthritis: An Updated Dose-Response Meta-Analysis

**DOI:** 10.1155/2019/3579081

**Published:** 2019-07-02

**Authors:** Xia Feng, Xizhu Xu, Yanjun Shi, Xuezhen Liu, Huamin Liu, Haifeng Hou, Long Ji, Yuejin Li, Wei Wang, Youxin Wang, Dong Li

**Affiliations:** ^1^Department of Epidemiology and Health Statistics, School of Public Health, Shandong First Medical University & Shandong Academy of Medical Sciences, Tai'an, China; ^2^Department of Rheumatology, Liaocheng People's Hospital, Liaocheng, China; ^3^Department of Epidemiology and Health Statistics, School of Public Health, Capital Medical University, Beijing, China

## Abstract

**Background:**

Extensive studies have been carried out to investigate the association between obesity and the risk of rheumatoid arthritis (RA); however, the results of the current reported original studies remain inconsistent. This study aimed to clarify the relationship between body mass index and rheumatoid arthritis by conducting an updated overall and dose-response meta-analysis.

**Methods:**

The relevant literature was searched using the PubMed and Embase databases (through 20 September 2018) to identify all eligible published studies. Random-effect models and dose-response meta-analyses were used to estimate the pooled risk ratio (RR) with a 95% confidence interval (CI). Subgroup analyses were also conducted based on the characteristics of the participants. Sensitivity analyses and publication bias tests were also performed to explore potential heterogeneity and bias in the meta-analysis.

**Results:**

Sixteen studies that included a total of 406,584 participants were included in the meta-analysis. Compared to participants with normal weight, the pooled RRs of rheumatoid arthritis were 1.12 (95% CI, 1.04-1.20) in overweight and 1.23 (95% CI, 1.09-1.39) in obese participants. There was evidence of a nonlinear relationship between body mass index (BMI) and RA (*P  *for nonlinearity less than 0.001 in the overall meta-analysis,* P* for nonlinearity=0.025 in the case-control studies,* P* for nonlinearity=0.0029 in the cohort studies). No significant heterogeneity was found among studies (*I*^2^=10.9% for overweight and *I*^2^=45.5% for obesity).

**Conclusion:**

The overall and dose-response meta-analysis showed that increased BMI was associated with an increased risk for rheumatoid arthritis, which might present a prevention strategy for the prevention or control of rheumatoid arthritis. The nonlinear relationship between BMI and RA might present a personal prevention strategy for RA.

## 1. Introduction

Rheumatoid arthritis (RA), the most common inflammatory arthritis, is a chronic systemic autoimmune inflammatory disease that is characterized by aggressive symmetric inflammation of multiple joints; RA affects 1-2% of adults, and the prevalence of RA is approximately 0.5-1.0% in the world [1–4]. Epidemiological investigations have documented that approximately 90% of RA patients develop bone erosions within 2 years of disease onset, which eventually leads to joint deformities or even disability [5]. Therefore, RA results in a heavy burden and great pain in the effected families, patients, and even the society as a whole. Although the etiology and pathogenesis of RA remain unclear, it is considered to be a multifactorial disease that results from the interaction between genetic and environmental factors [6]. It has been reported in a number of studies that a well-established environmental risk factor that is associated with increased RA risk is tobacco smoking [7–9]. However, other factors affecting rheumatoid arthritis need to be further explored.

Obesity, defined as an increase in fat at a sufficient level to cause adverse health consequences, is usually diagnosed by anthropometric measurements of body mass index (BMI), which is calculated as weight in kilograms divided by the square of height in meters (kg/m^2^) [10]. As a pandemic public health issue in the western world, overweight/obesity is associated with a high incidence of chronic autoimmune and inflammatory pathologies, such as type 2 diabetes and rheumatoid arthritis, thus resulting in a large social and economic impact [11]. According to previous statistics, more than 60% of patients with RA are classified as overweight or obese by body mass index (BMI≥25 kg/m^2^) [12, 13]. In addition, obesity represents an important and increasingly prevalent comorbidity even at the first presentation of RA [14]. Although the relationship between obesity and RA has been widely reported in previous original studies and meta-analyses, inconsistent results have also been implied in recent studies. For example, Ljung et al. reported that obesity was associated with an increased risk of RA, and this finding appeared to be primarily associated with early-onset RA in men [15], while Turesson et al. reported that a high BMI was associated with a reduced risk of future RA in men but not in women [16]. Considering that the new original published articles may present different information than previous research results, a new meta-analysis is necessary to clarify the relationship between BMI and RA. Therefore, we conducted this updated overall and dose-response meta-analysis to further explore the association between overweight/obesity and RA.

## 2. Materials and Methods

### 2.1. Literature Search

We electronically searched the PubMed and Embase databases for studies published through 20 September 2018. The comprehensive search strategies included the MeSH terms and keywords of “overweight” or “obesity” or “obese” or “body mass index” or “BMI” combined with “rheumatoid arthritis.” Observational studies on BMI and rheumatoid arthritis were included in our meta-analysis, without any restrictions of language and ethnicity. As we conducted an updated systematic review, we reviewed the published meta-analysis by Qin et al. [17] to further identify eligible relevant articles. We also reviewed the references from other relevant studies.

### 2.2. Study Selection

Studies that met the following criteria were included in our meta-analysis: (1) a case-control study or a cohort study; (2) overweight, obesity, and BMI were the exposures of interest; (3) rheumatoid arthritis (RA) was the outcome of interest; (4) the study reported the relative risk (RR) or odds ratio (OR) with the corresponding 95% confidence intervals (95% CIs) or sufficient data to calculate them for the association between BMI and RA risk; and (5) when the studies had overlapping populations, only studies with the most detailed information or the largest sample size were included. For a dose-response meta-analysis, the BMI must be separated into three or more categories, so the RA risk corresponding to each category could be estimated. Studies that did not provide sufficient or original data were excluded. Reviews, case reports, mechanism studies, unpublished studies, and nonhuman studies were also excluded.

### 2.3. Data Extraction

Data were extracted independently by two investigators and checked for accuracy by a 3rd investigator. The following variables were obtained from each qualified publication: first author's name, publication year, country, study design, age of participants, gender of participants, study sample size, measurement of BMI (self-reported or measured by investigator), BMI categories, the number of cases or person-year data in each BMI category, and the adjusted RR or OR and its 95% CI. To reduce the impact of confounding factors, we used RR and OR to adjust for covariates in multivariate models. Study quality was assessed using the Newcastle-Ottawa Scale (NOS), which included 8 items and provided a numeric quality score ranging from 0 to 9 stars [18].

### 2.4. Statistical Analysis

The median or mean BMI for each category was assigned the corresponding RR or OR. If the median or mean BMI of each category was not reported in the study, the midpoint of the upper and lower limits of each BMI category was defined as the average. When the upper and lower limits of the highest and lowest categories were not provided, we assumed that the limits had the same amplitude as that of the adjacent category. For studies that provided only the total number of cases and person-years, we used the method of Aune et al. [19] to estimate the stratified number of cases and person-years in each group. According to the World Health Organization (WHO) guidelines, we classified BMI (kg/m^2^) into three categories: normal weight (18.50 to 24.99), overweight (25.00 to 29.99), and obesity (>30.00) [20]. We used a random-effect model to compare the risk between overweight/obesity and normal BMI and to estimate the summary RRs and/or ORs with their 95% CI [21]. To investigate the effect of potential confounders, subgroup analyses were conducted with the available characteristics of studies and participants if three or more studies were available per subgroup. Considering the heterogeneity between studies, we performed a two-stage random-effect dose-response meta-analysis to calculate the trend based on relevant logRRs estimated across levels of BMI [22]. First, a generalized least squares regression was used to estimate the restricted cubic spline model distributed at the 10th, 50th, and 90th percentiles, taking into account the correlation within each set of reported RRs and/or ORs. Then, we used the method described by Greenland and Longnecker to conduct a dose-response meta-analysis, which required cases, person-years, and doses converted from BMI, as well as BMI category-specific RRs and/or ORs with variance estimated for at least three quantitative classifications of each article [23, 24]. Additionally, the study-specific estimates were combined using the restricted maximum likelihood method in a multivariate random-effects meta-analysis [25]. The P value for nonlinearity was calculated by testing the null hypothesis that the coefficient of the second spline was equal to zero [26]. A linear model was used to estimate the linear trends of RRs and/or ORs for RA per 5 kg/m^2^ increase in BMI if there was no evidence to prove the nonlinear relationship. We judged the heterogeneity between studies with the Q-test and *I*^2^ statistic, and *I*^2^ values of 0%, 25%, 50%, and 75% represented no, low, moderate, and high heterogeneity, respectively [27]. We also conducted subgroup analyses and meta-regression analyses to further investigate the potential sources of heterogeneity and whether the relationship between BMI and RA was biased by study-specific factors (e.g., age, sex, smoking, alcohol, location, and assessment method of BMI). In addition, a sensitivity analysis was performed to estimate the stability of our meta-analysis. To conduct the sensitivity analysis, one study was removed at a time and the remaining studies were analyzed to clarify whether the results were markedly affected by a single study. The publication bias was assessed by inspecting the funnel plots for asymmetry and with Egger's test [28]. All statistical analyses were performed with Stata 12.0 (Stata Corporation, College Station, TX). All reported *P* values were two-sided, with* P* < 0.05 considered statistically significant.

## 3. Results

### 3.1. Literature Search and Study Characteristics

**Figure 1:**
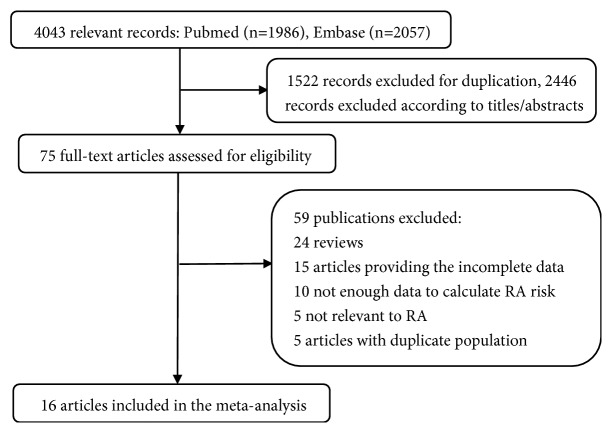
Flowchart of the selection of studies for inclusion in this meta-analysis.

A total of 4,043 records were identified through September 20, 2018, from the two aforementioned databases. After excluding 1,522 duplicate records, 2,466 irrelevant records were excluded after screening the titles and abstracts. After detailed evaluation, a total of 406,584 participants in 16 studies [15, 16, 29–42] were included in our meta-analysis, as shown in Figure 1. There were 11 case-control studies that did not have a time-sequence relationship between BMI and RA [15, 16, 29–37], and 5 cohort studies in which BMI was measured before RA [38–42].

The general characteristics of the studies included in this meta-analysis are shown in Table 1. In addition to the 11 original studies analyzed by Qin et al. [17], our literature search identified 5 additional studies [15, 16, 36–38], out of which 4 studies [15, 16, 36, 37] were published after Qin's meta-analysis. Among the included studies, 10 studies [15, 16, 30–34, 38, 40, 41] were conducted in Europe, 4 studies [29, 35, 39, 42] in North America, and 2 studies [36, 37] in Asia. Four studies [29, 39, 40, 42] and one study [38] only reported separated outcomes in women and men, respectively, while 11 studies [15, 16, 30–37, 41] reported outcomes in both sexes. Of the 11 studies, 4 studies [15, 16, 32, 34] reported outcomes in men and women separately, and 7 studies [30, 31, 33, 35–37, 41] combined the data of both sexes.

### 3.2. BMI and Rheumatoid Arthritis Risk

**Figure 2:**
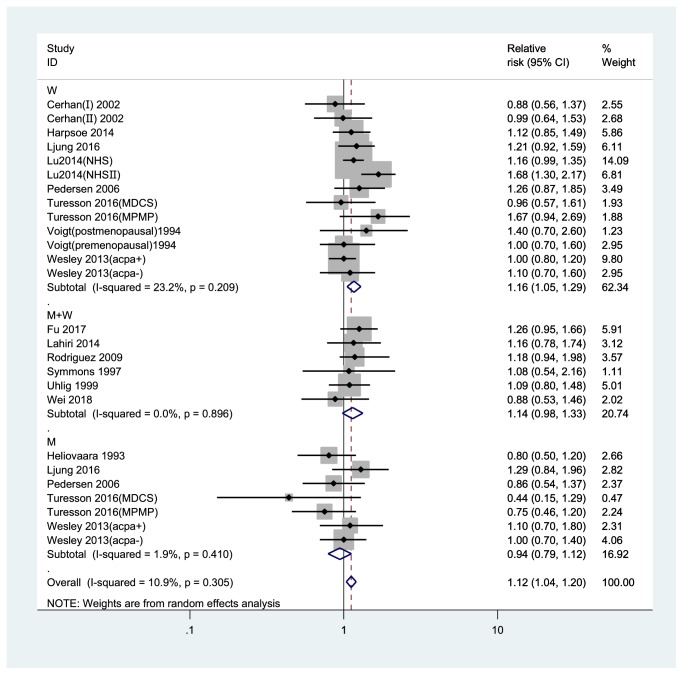
*Forest plot of the RRs of overweight vs. normal weight individuals for rheumatoid arthritis risk*. RR, relative risk; CI, confidence interval; BMI, body mass index; M, men; W, women.

**Figure 3:**
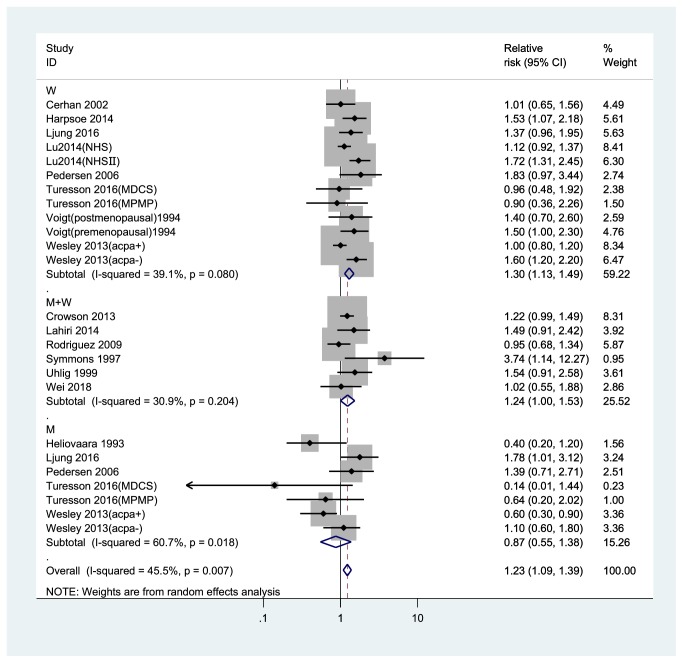
*Forest plot of the RRs of obesity vs. normal weight individuals for rheumatoid arthritis risk*. RR, relative risk; CI, confidence interval; BMI, body mass index; M, men; W, women.

Compared to the normal weight group, the pooled RRs of RA were 1.12 (95% CI, 1.04-1.20) in overweight and 1.23 (95% CI, 1.09-1.39) in obese participants (Figures 2 and 3). No significant heterogeneity was found among studies (overweight: *I*^2^= 10.9%; obesity: *I*^2^= 45.5%).

### 3.3. Subgroup Analysis

For the category of overweight and obesity, a subgroup meta-analysis revealed a result that was mostly consistent with the results of the overall analysis (Table 2). The RR associated with overweight among women was 1.16 (95%CI, 1.05-1.29) and the RR associated with obesity among women was 1.30 (95%CI, 1.13-1.49), while the risk of RA for men was not significant in the overweight (0.94 (95% CI, 0.79-1.12)) or obese (0.87 (95% CI, 0.55-1.38)) categories. The pooled association of RA with overweight and obesity compared to normal BMI was statistically significant for both overweight and obesity in studies with NOS quality scores ≥7 (RR, 1.12; 95% CI, 1.04-1.21 and RR, 1.21; 95% CI, 1.05-1.39 for overweight and obesity, respectively), in case-control studies (OR, 1.09; 95% CI, 1.00-1.19 and OR, 1.22; 95% CI, 1.05-1.31 for overweight and obesity, respectively), in studies that adjusted for age (RR, 1.12; 95% CI, 1.04-1.21 and RR, 1.23; 95% CI, 1.08-1.39 for overweight and obesity, respectively), in studies that adjusted for smoking (RR, 1.12; 95% CI, 1.02-1.22 and RR, 1.24; 95% CI, 1.08-1.42 for overweight and obesity, respectively), while only the pooled association of RA for obesity versus normal weight was statistically significant in European studies (RR, 1.20; 95% CI, 1.00-1.43), in North America studies (RR, 1.27; 95% CI, 1.10-1.48), in self-reported studies (RR, 1.28; 95% CI, 1.09-1.52), in studies not adjusting alcohol consumption studies (RR, 1.27; 95% CI, 1.08-1.49).

### 3.4. Dose-Response Meta-Analysis

**Figure 4:**
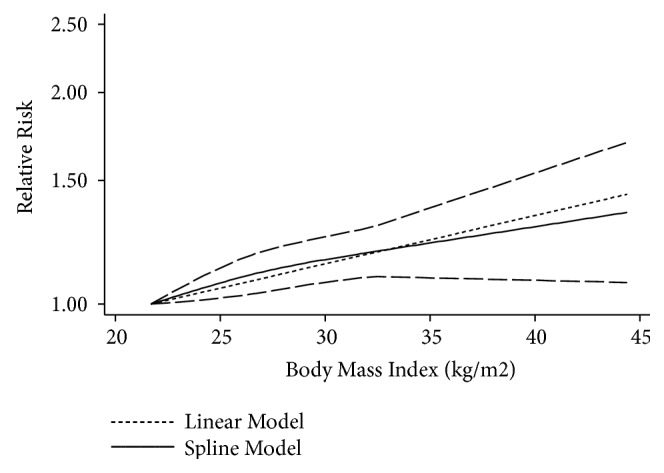
*The dose-response analysis between BMI and rheumatoid arthritis risk in studies with restricted cubic splines in a multivariate random-effects dose-response model*. The solid line and the long dashed line represent the estimated RR and its 95% CI. The short dashed line represents the linear relationship (per 5 kg/m^2^ increment). RR, relative risk; CI, confidence interval; BMI, body mass index.

**Figure 5:**
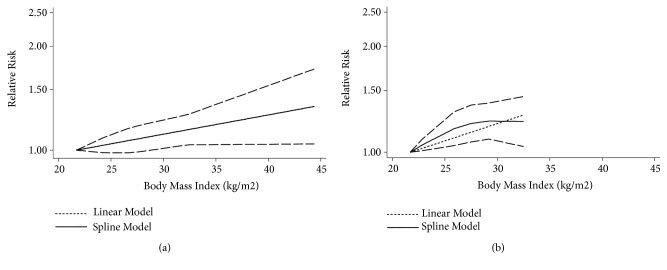
*The dose-response analysis for case-control studies and cohort studies between BMI and rheumatoid arthritis risk*. (a) Case-control studies; (b) cohort studies. The solid line and the long dashed line represent RR and its 95% CI. The short dashed line represents the linear relationship (per 5 kg/m^2^ increment). RR, relative risk, CI, confidence interval; BMI, body mass index.

A total of 12 studies [15, 16, 29–34, 38, 39, 42] were included in our dose-response meta-analysis. The linear association showed an increased RA risk of 1.08 (95% CI, 1.01-1.15) for each 5 kg/m^2^ increase in BMI. In addition, there was evidence indicating a nonlinear relationship between BMI and RA (*P* for nonlinearity less than 0.001 in the overall meta-analysis, *P* for nonlinearity=0.025 in the case-control studies, *P* for nonlinearity=0.0029 in the cohort studies). Compared to participants with a BMI of 21.4 kg/m^2^, the summary RRs (95% CIs) of RA were 1.09 (1.03-1.16), 1.15 (1.07-1.23), 1.19 (1.09-1.29), and 1.35 (1.07-1.70) in participants with BMIs of 25, 30, 35, and 40 kg/m^2^, respectively (Figure 4). Considering that differences in study designs may lead to differences in research results, we performed a dose-response meta-analysis stratified by study design. In a meta-analysis of 9 case-control studies [15, 16, 29–34, 37], the summary ORs (95% CIs) of RA were 1.06 (0.98-1.15), 1.10 (1.00-1.21), 1.15 (1.04-1.27), and 1.34 (1.04-1.72) for BMIs of 25, 30, 35, and 40 kg/m^2^, respectively, compared to a BMI of 21.3 kg/m^2^ (Figure 5(a)). In a meta-analysis of 3 cohort studies [38, 39, 42], the corresponding summary RRs (95% CIs) of RA were 1.17 (1.04-1.30) and 1.23 (1.09-1.38) for BMIs of 25 and 30 kg/m^2^, respectively, compared to a BMI of 21.7 kg/m^2^(Figure 5(b)).

### 3.5. Publication Bias

**Figure 6:**
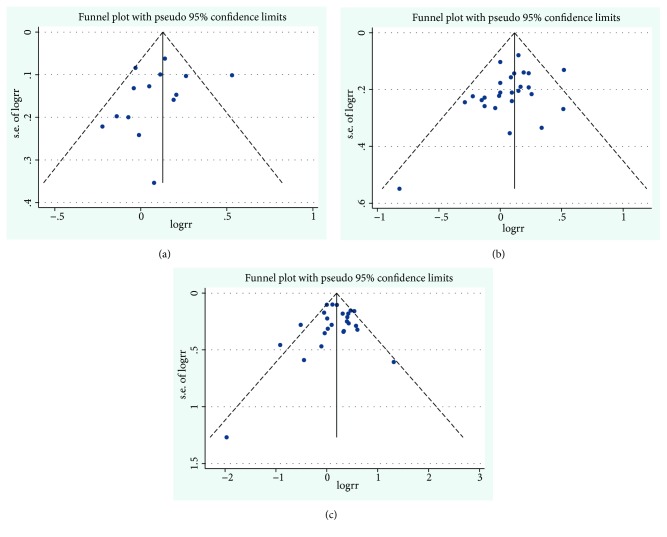
*Funnel plot corresponding to the random-effects meta-analysis of the relationship between BMI and rheumatoid arthritis risk*. (a) Funnel plot corresponding to the dose-response meta-analysis of the relationship between BMI and rheumatoid arthritis risk (P_Egger's  test_ = 0.334); (b) overweight and rheumatoid arthritis risk (P_Egger's  test_= 0.110); and (c) obesity and rheumatoid arthritis risk (P_Egger's  test_= 0.781). BMI, body mass index.

The funnel plots for the pooled RRs of RA risk are shown in Figure 6. Egger's test showed that there was no publication bias in the literature on BMI and RA risk in the dose-response group (P_Egger's  test_ = 0.334,), in the overweight group (P_Egger's  test_= 0.110) or in the obesity group (P_Egger's  test_= 0.781).

### 3.6. Sensitivity Analysis

In a sensitivity analysis in which one study at a time was removed and the rest were analyzed, the pooled RRs ranged from 1.04 to 1.20 for overweight, from 1.09 to 1.39 for obesity, and from 1.01 to 1.24 for the dose-response analysis, which demonstrated that the pooled estimates were stable.

## 4. Discussion

In our meta-analysis, we found a 12% increased risk of RA in overweight participants and a 23% increased risk in obese participants compared with normal weight participants. When the analysis was stratified by sex, the RA risk for obese versus normal weight participants was higher in women compared to the risk for both sexes combined. The dose-response meta-analysis revealed that each 5 kg/m^2^ increase in BMI resulted in an 8% increase in the risk of RA. In addition, a significant nonlinear relationship between BMI and RA was found in the overall studies, as well as in case-control studies and cohort studies.

A previous meta-analysis by Qin et al. [17] reported a positive association between overweight/obesity and RA compared to the association in normal weight individuals. In addition, Qin et al. explored the nonlinear relationship between BMI and RA in overall studies and did not stratify by study design. Feng et al. [43] also reported a positive association between BMI and RA only in women, and the increased risk of RA had a linear relationship for every 5 kg/m^2^ increase in BMI. Our results, which are more comprehensive and based on 16 studies, were generally in line with the results from the previous meta-analyses [17, 43]. In addition, we also found a significant nonlinear relationship between BMI and RA, in both case-control studies and cohort studies. Specifically, compared to participants with a BMI of 21.7 kg/m^2^, those with a BMI ranging from 21.8 to 25.0 and from 25.1 to 30.0 kg/m^2^ had a 17% and 6% increased risk of RA, respectively, in the cohort study. The nonlinear relationship between BMI and RA remains unexplained. In addition, our subgroup analysis showed that the risks of RA were different in studies which adjusted for age or not adjusted for age, adjusted for smoking or not adjusted for smoking, and adjusted for alcohol or not adjusted for alcohol. The results suggested that the association between BMI and risk of RA might be interfered by age, smoking, and alcohol, so the future epidemiological studies should acknowledge them.

The present study showed that obesity or a higher BMI increases the risk of RA. This finding was consistent with a Mendelian randomization study, which included a study of 337,159 individuals and demonstrated that BMI was causally associated with an increased risk of RA [44]. However, the mechanism underlying the association remains unclear. The adipose tissue of obese individuals secretes inflammatory cytokines such as leptin, TNF-*α*, IL-6, interleukin-1*β*, and monocyte chemotactic protein-1 (MCP-1) [45]. These adipokines induce an inflammatory response in individuals [46]. Previous studies have shown elevated levels of these inflammatory markers in individuals before the onset of RA [47]. Obesity has been significantly related to concentrations of several sex hormones, such as estrogen, estradiol, and free estradiol [48]. In addition, estrogen has been suggested to play a role as an immunomodulator [49]. Several previous studies have indicated that sex hormones can act on a variety of immune cells (e.g., T cells, B cells, and monocytes) and interfere with the expression and production of proinflammatory cytokines, thereby affecting the development of RA [50–52].

The strengths of our study were the inclusion of more literature and a large number of participants, which made the results more reliable. Moreover, we conducted subgroup analyses to explore the relationship between BMI and RA, which controlled the effects of possible confounding factors as much as possible. In addition, the potential nonlinear relationship between BMI and RA was assessed not only in overall studies but also in case-control studies and cohort studies separately.

Although this meta-analysis included more studies than the previous meta-analyses, it has several potential limitations. First, most of the included studies were case-control studies, and in most studies, BMI was self-reported by participants rather than assessed by medical measurements, which might lead to biased results. Second, since we only searched the two databases PubMed and Embase, our results might be generalized mainly to Europeans/Americans, and the potential publication bias was inevitable because our meta-analysis only included published studies. Third, we had performed multiple subgroup analyses to control for potential confounders. The strategy might increase the risk for type I error and the risk for false-positive findings, particularly in the setting low statistical heterogeneity. Finally, we neglected to register the protocol before the formal conduct.

## 5. Conclusions

To summarize, the dose-response meta-analysis systematically evaluated the relationship between BMI and RA. The results confirmed that increased BMI was associated with an increased risk of RA. Understanding the association between BMI and RA might benefit the prevention or control of RA.

## Figures and Tables

**Table 1:** Characteristics of included studies.

Author (year)	country	study design	Age	Gender	NOS	Sample size	Method of weight/height	BMI category	OR(RR)(95%CI)		Adjustment factors
						RA/Control		(kg/m^2^)	Men	Women	
Voigt, 1994	USA	Case-control	18-64	Women	7	349/1457	Self-reported	premenopausal	NA		Age, smoking status
								12.94-20.43		1.00	
								20.44-22.51		1.10(0.70-1.60)	
								22.52-25.82		1.00(0.70-1.60)	
								25.83-52.86		1.50(1.00-2.30)	
								postmenopausal			
								12.94-20.43		1.00	
								20.44-22.51		1.10(0.50-2.10)	
								22.52-25.82		1.40(0.70-2.60)	
								25.83-52.86		1.40(0.70-2.60)	

Symmons, 1997	UK	Case-control	18-70	Mixed	7	90/93	Self-reported	20.0-24.9	Mixed	1.00	Smoking status, social class
								25.0-29.9		1.08(0.54-2.16)	
								30.0 -40.0		3.74(1.14-12.27)	

Uhlig, 1999	Norway	Case-control	20-79	Mixed	7	347/5725	Self-reported	<25.0	Mixed	1.00	Age, sex, marital status, employment category, formal education, current smoking status.
								25.0-29.9		1.09(0.80-1.48)	
								≥30.0		1.54(0.91-2.58)	

Pedersen, 2006	Denmark	Case-control	18-65	Mixed	6	505/752	Self-reported	<18.5	0.95(0.19-4.72)	0.95(0.52-1.73)	Birth year, year of RA diagnosis, gender
								18.5-25.0	1.00	1.00	
								25.0-30.0	0.86(0.54-1.37)	1.26(0.87-1.85)	
								>30.0	1.39(0.71-2.71)	1.83(0.97-3.44)	

Rodriguez, 2009	UK	Case-control	20-79	Mixed	8	559/4234	Measured	<20.0	Mixed	0.65(0.43-0.98)	Age, sex, calendar year, number of referrals, and visits to a primary care physician
								20.0-24.9		1.00	
								25.0-30.0		1.18(0.94-1.98)	
								>30		0.95(0.68-1.34)	

Wesley, 2013	Sweden	Case-control	18-70	Mixed	7	2748/3444	Self-reported	ACPA-Positive			Sex, age, area of residence, smoking, alcohol consumption, education
								<25.0	1.00	1.00	
								25.0-30.0	1.10(0.70-1.80)	1.00(0.80-1.20)	
								≥30.0	0.60(0.30-0.90)	1.00(0.80-1.20)	
								ACPA-Negative			
								<25.0	1.00	1.00	
								25.0-30.0	1.00(0.70-1.40)	1.10(0.70-1.60)	
								≥30.0	1.10(0.60-1.80)	1.60(1.20-2.20)	

Crowson, 2013	USA	Case-control	≥18	Mixed	5	813/813	Measured	<30.0	Mixed	1.00	Age, sex and calendar year, smoking status
								≥30.0		1.22(0.99-1.49)	

Ljung, 2016	Sweden	Case-control	25-74	Mixed	7	557/1671	Measured	<25.0	1.00	1.00	Smoking habits, educational level, age, sex, year of health examination, cohort
								25.0-30.0	1.29(0.84-1.96)	1.21(0.92-1.59)	
								≥30.0	1.78(1.01-3.12)	1.37(0.96-1.95)	

Turesson, 2016	Sweden	Case-control	58 ± 7.2	Mixed	8	MDCS (172/688)	Measured	MDCS			Smoking, level of formal education, alcohol consumption, socio-economic status
								18.5-25.0	1.00	1.00	
								25.0-30.0	0.44(0.15-1.29)	0.96(0.57-1.61)	
								>30.0	0.14(0.01-1.44)	0.96(0.48-1.92)	
						MPMP (290/1160)		MPMP			
								18.5-25.0	1.00	1.00	
								25.0-30.0	0.75(0.46-1.20)	1.67(0.94-2.69)	
								>30.0	0.64(0.20-2.02)	0.90(0.36-2.26)	

Fu, 2017	China	Case-control	18-75	Mixed	7	400/400	Self-reported	<23.9	Mixed	1.00	Sex
								>24.0		1.26(0.95-1.66)	

Wei, 2018	China	Case-control	30-75	Mixed	7	403/128	Self-reported	<25.0	Mixed	1.00	Age, sex, waist circumference, erosive osteoarthritis, duration of disease, current smoker,
								25.0-30.0		0.88(0.53-1.46)	
								≥30.0		1.02(0.55-1.88)	

Heliovaara, 1993	Finland	Cohort	30-69	Men	9	28364	Measured	<25.0	1.00	NA	Smoking, geographical region, type of population, marital status, social class, perceived, health, and age
								25.0-30.0	0.80(0.50-1.20)		
								>30.0	0.40(0.20-1.20)		

Cerhan, 2002	USA	Cohort	55-69	Women	7	31336	Self-reported	<23.4	NA	1.00	Age
								23.4-25.8		0.88(0.56-1.37)	
								25.9-29.2		0.99(0.64-1.53)	
								>29.2		1.01(0.65-1.56)	

Harpsoe, 2014	Denmark	Cohort	27.4-33.3	Women	7	75008	Self-reported	<18.5	NA	0.82(0.45-1.50)	Smoking status, alcohol consumption, parity, socio-occupational status
								18.5-25.0		1.00	
								25.0-30.0		1.12(0.85-1.49)	
								≥30.0		1.53(1.07-2.18)	

Lahiri, 2014	UK	Cohort	40-79	Mixed	9	25455	Self-reported	<25.0	Mixed	1.00	Age, gender, smoking status, breastfeeding, alcohol consumption
								25.0-30.0		1.16(0.78-1.74)	
								>30.0		1.49(0.91-2.42)	

Lu, 2014	USA	Cohort	NHS: 30-55	Women	7	NHS:109896	Measured	NHS			Age, community median income, smoking status, alcohol consumption, physical activity, parity, breastfeeding status, postmenopausal use, postmenopausal Hormone use
								18.5-24.9	NA	1.00	
								25.0-29.9		1.16(0.99-1.35)	
								≥30.0		1.12(0.92-1.37)	
			NHSII: 25-42			NHS2:108727		NHSII			
								18.5-24.9	NA	1.00	
								25.0-29.9		1.68(1.30-2.17)	
								≥30.0		1.72(1.31-2.45)	

BMI, body mass index; RR, relative risk; CI, confidence interval; NA, not available; M, men; W, women; NOS, New castale-Ottawa Scale; NHS, Nurses' Health Study.

**Table 2:** Subgroup analyses of BMI and rheumatoid arthritis risk.

Study	overweight			obesity		
	No. of studies	OR RR (95%CI)	I^2^(%)	No. of studies	OR RR (95%CI)	I^2^(%)
All studies	15	1.12(1.04-1.20)	10.9	15	1.23(1.09-1.39)	45.5
*Sex*						
Men	5	0.94(0.79-1.12)	1.9	5	0.87(0.55-1.38)	60.7
Women	8	1.16(1.05-1.29)	23.2	8	1.30(1.13-1.49)	39.1
Combined	6	1.14(0.98-1.33)	0	6	1.14(0.98-1.33)	0
*Study location*						
Asia	2	1.12(0.81-1.56)	32.4	1	1.02(0.55-1.89)	0
Europe	10	1.07(0.98-1.17)	0	10	1.20(1.00-1.43)	53.3
North America	3	1.18(0.97-1.45)	51.7	4	1.27(1.10-1.48)	28.3
*NOS*						
<7	1	1.07(0.74-1.55)	35.9	2	1.28(1.06-1.54)	0
≥7	14	1.12(1.04-1.21)	13	13	1.21(1.05-1.39)	50.3
*type*						
*case-control*	10	1.09(1.00-1.19)	0	10	1.22(1.05-1.40)	39.4
*cohort*	5	1.14(0.96-1.35)	53.1	5	1.24(0.96-1.61)	64.3
*Assessment method of weight/height*						
Self-reported	10	1.07(0.97-1.17)	0	9	1.28(1.09-1.52)	44.7
Measured	5	1.15(0.97-1.37)	53.8	6	1.16(0.96-1.40)	50.9
*Adjustment factors*						
*Age*						
Yes	11	1.12(1.04-1.21)	6.3	12	1.23(1.08-1.39)	47.1
No	4	1.09(0.88-1.34)	32.1	3	1.15(0.70-1.90)	48.6
*Smoking*						
Yes	11	1.12(1.02-1.22)	21.6	12	1.24(1.08-1.42)	49.4
No	4	1.11(0.95-1.30)	0	3	1.13(0.86-1.49)	21.4
*Alcohol consumption*						
Yes	5	1.13(0.99-1.28)	43.5	5	1.18(0.98-1.42)	57.1
No	10	1.10(0.99-1.22)	0	10	1.27(1.08-1.49)	33.7

BMI, body mass index; RR, relative risk; CI, confidence interval.
